# Respiratory Condition of Family Farmers Exposed to Pesticides in the State of Rio de Janeiro, Brazil

**DOI:** 10.3390/ijerph15061203

**Published:** 2018-06-08

**Authors:** Rafael J. Buralli, Helena Ribeiro, Thais Mauad, Luís F. Amato-Lourenço, João M. Salge, Fredi A. Diaz-Quijano, Renata S. Leão, Rejane C. Marques, Daniele S. Silva, Jean Remy Davée Guimarães

**Affiliations:** 1 Departamento de Saúde Ambiental, Faculdade de Saúde Pública, Universidade de São Paulo, Av. Dr Arnaldo, 715, São Paulo, SP 01246-904, Brazil; lena@usp.br; 2 Departamento de Patologia, Faculdade de Medicina, Universidade de São Paulo, Av. Dr Arnaldo, 455, sala 1155, São Paulo, SP 01246-903, Brazil; tmauad@usp.br (T.M.); luisfamato@gmail.com (L.F.A.-L.); 3 Pneumologia, Hospital das Clínicas, Faculdade de Medicina, Universidade de São Paulo, Av. Dr Enéas Carvalho de Aguiar, 44-Bloco II, 5 andar, São Paulo, SP 05403000, Brazil; joao.salge@incor.usp.br; 4 Departamento de Epidemiologia, Faculdade de Saúde Pública, Universidade de São Paulo, Av. Dr Arnaldo, 715, São Paulo, SP 01246-904, Brazil; frediazq@msn.com; 5 Centro de Tecnologia em Nanomateriais—CTNANO, Rua Prof. José Vieira de Mendonça, 1000, Belo Horizonte, MG 31310-260, Brazil; rspolti@hotmail.com; 6 Universidade Federal do Rio de Janeiro—Campus Macaé, Av. Aloísio da Silva Gomes, 50, Macaé, RJ 27930-560, Brazil; rejanecmarques@globo.com; 7 Instituto de Biofísica Carlos Chagas Filho, Universidade Federal do Rio de Janeiro, Av. Carlos Chagas Filho 373-Bloco G-CCS, Ilha do Fundão, Rio de Janeiro, RJ 21941-902, Brazil; silva.danielesantos@gmail.com (D.S.S.); jeanrdg@biof.ufrj.br (J.R.D.G.)

**Keywords:** pesticides, spirometry, respiratory symptoms, cholinesterase, rural workers, family farmers

## Abstract

Pesticide exposure is a growing public health concern. Although Brazil is the world’s largest consumer of pesticides, only a few studies have addressed the health effects among farmers. This study aimed to evaluate whether pesticide exposure is associated with respiratory outcomes among rural workers and relatives in Brazil during the crop and off-seasons. Family farmers (82) were interviewed about occupational history and respiratory symptoms, and cholinesterase tests were conducted in the crop-season. Spirometry was performed during the crop and off-season. Respiratory outcomes were compared between seasons and multiple regressions analysis were conducted to search for associations with exposure indicators. Participants were occupationally and environmentally exposed to multiple pesticides from an early age. During the crop and off-season, respectively, they presented a prevalence of 40% and 30.7% for cough, 30.7% and 24% for nasal allergies, and 24% and 17.3% for chest tightness. Significant associations between spirometry impairments and exposure indicators were found both during the crop and off-season. These findings provide complementary evidence about the association of pesticide exposure with adverse respiratory effects among family farmers in Brazil. This situation requires special attention as it may increase the risk of pulmonary dysfunctions, and the morbidity and mortality burden associated with these diseases.

## 1. Introduction

Careless pesticide use is a major human health problem, particularly in low and middle-income countries where public policies tend to be less restrictive and health surveillance less effective [1,2]. In Brazil, agriculture plays a crucial role in the economic development, and since 2008, the country has been the world’s largest consumer of pesticides [1]. Brazilian family farmers are often exposed to large amounts of pesticides due to the low risk-awareness and educational level, lack or misuse of personal protective equipment (PPE), lack of technical support, frequent use of highly toxic compounds, proximity of households and application sites, and relatives working or helping in different cultivation tasks, among others [1,3,4].

Occupational exposure to pesticides can represent a serious risk to the respiratory system [5,6,7]. Epidemiological studies have linked it to respiratory symptoms [8,9,10], asthma [11,12,13], chronic bronchitis [9,14,15,16], and lung cancer [7]. Spirometry was performed in workers occupationally exposed to pesticides and revealed a significant decrease in the lung function parameters both in cross-sectional studies comparing with non-exposed controls [8,9,17,18] and in prospective cohort studies [16,19,20]. Only few cross-sectional studies were conducted with pesticide-exposed rural workers in Brazil and reinforce the findings regarding its effects on the respiratory symptoms [3,21,22]. One study, published in 2005, evaluated the lung function of rural workers in Brazil and found a high prevalence of ventilatory disorders [23].

Assessing the individual exposure to pesticides is a main challenge in studies with occupationally exposed communities. Biological monitoring is often used to estimate the extent of exposure and establish causal relations with health outcomes. Despite all concerns about its specificity, sensitivity, and individual and laboratory variations, the most common test used in Brazil is the quantification of acetylcholinesterase (AChE) and butyrylcholinesterase (BChE) activity, which are inhibited by organophosphorus (OF) and carbamate (CM) pesticides [1,4].

Therefore, the present study aimed to explore whether exposure to pesticide is associated with the prevalence of respiratory symptoms and lung function impairments among workers and their families in small rural properties in Brazil during both crop season and off-season, using cholinesterase exams, among other research instruments. These data can be useful to better understand the pesticide exposure scenario in family farming and to inspire public policies to face the problem. Moreover, they can raise awareness among Brazilian family farmers to improve their work practices and policy makers to provide more training and technical support.

## 2. Materials and Methods

This study was carried out in two stages: (a) during the crop season (July and August 2014), questionnaire-based interviews about sociodemographic characteristics, clinical information and detailed exposure history to pesticide were conducted, biomarkers were collected for analysis, and the respiratory assessment was performed; (b) during the off-season period (January 2015), all participants underwent the respiratory assessment again to compare higher and lower exposure periods. This study was approved by the Ethical Board of the University Hospital Clementino Fraga Filho of the Federal University of Rio de Janeiro, and all participants provided written informed consent.

### 2.1. Study Area and Population

São José de Ubá (SJU, Brazil) is a small town located in the northwest of Rio de Janeiro State, Brazil. It has approximately 7000 inhabitants, 45% of whom live in the urban center while 55% are distributed in rural neighborhoods of 200 to 300 people. The economy is mostly based on family farming, especially tomato cultivation [24], which demands intensive phytosanitary care for pest control, usually based on the use of significant amounts of pesticides [25]. Studies previously conducted in the area evaluated the quality of surface and groundwater and found nitrate, aluminum, iron, manganese, boron, zinc and pesticides (organochlorine and OF) in disagreement with the levels allowed by Brazilian legislation as a result of agricultural practices, livestock and untreated sewage disposal [26,27].

The sample in our study consisted of 82 individuals older than 18 years from approximately 750 individuals working in tomato cultivation in SJU. Participants were rural workers (*n* = 48) or relatives (*n* = 34) residing in the rural area. Rural workers were those daily involved in tomato cultivation at the time the study was conducted, which included pesticide handling. Relatives were those members of the same family (relatives that lived in the same household), which could help in agricultural-related activities. Recruitment of participants was done by convenience in agricultural areas upon indication of SJU residents and stakeholders. Individuals were contacted in the rural properties and invited to participate. Participants were sought for reevaluation in the off-season period. The sample was obtained sequentially, including all eligible subjects that could be contacted during the study period. Thus, the final sample size was delimited by the projects’ time and budget constraints. Demographic data such as age, gender and body mass index (BMI) were obtained from each subject. Socio-economic status, educational level, smoking habits (pack-years), marital status and alcohol consumption data were also collected. The Brazilian minimum wage (R$954 Brazilian reais in 2018), which is equivalent to approximately $260 US dollars, was used as the basis to calculate the monthly family income.

### 2.2. Exposure Assessment

Exposure assessment was obtained through a questionnaire-based interview conducted by a trained researcher during the crop season. Information related to the duration of pesticide exposure, manipulation frequency, use of personal protective equipment (PPE), pesticide use in the off-season, domestic exposure, intoxication history, and hygiene habits after pesticide manipulation (washing hands and taking a shower after work or eating at the crop field) were obtained. It was asked the types of pesticides most frequently used by rural workers and they were classified according to the Brazilian National Sanitary Surveillance Agency (ANVISA): class I (extremely toxic), II (highly toxic), III (moderately toxic) and IV (low toxicity) [1]. This pesticide toxicity classification considers the acute oral LD_50_, dermal LD_50_, and inhalation LD_50_, ocular and cutaneous lesions tested in laboratory animals [28]. It was also assessed whether rural workers and relatives received technical orientation or training in safety procedures.

Based on previous studies [18,20,29,30], an individual exposure burden (IEB) was created with a range of 0–10, using: current contact with pesticides (no = 0/yes = 2); domestic exposure, such as manipulation of contaminated clothes and domestic use for pest control (no = 0/yes = 1); previous intoxication after pesticide exposure (no = 0/yes = 1); frequency of pesticides manipulation (no contact = 0, once a month or less = 1, 2–3 times/month = 2, 1–3 times/week = 3 or 5–7 times/week = 4); and distance from home to crop areas (more than 1 km = 0, from 500 m to 1 km = 1 or up to 500 m = 2). 

Regarding the cholinesterase activity measurement, blood samples (10 mL) were collected from 74 individuals by qualified personnel using heparinized Vacutainer tubes during the crop season. Samples were immediately centrifuged, frozen and sent to the Centro de Estudos da Saúde do Trabalhador e Ecologia Humana (CESTEH—Human Study Center for Worker’s Health and Human Ecology) from the National School of Public Health, Oswaldo Cruz Foundation (FIOCRUZ, Rio de Janeiro, Brazil) for analysis. Cholinesterase activity (AChE and BChE) was quantified by using a Shimadzu UV/VIS 1601 spectrophotometer, through the Ellman method, modified by Oliveira-Silva et al. [31]. This method is indicated when blood sampling is performed far from the laboratory and allows cholinesterase determination after freezing of plasma and erythrocyte fractions. Obtained values were compared to the exposure indicators and to reference values determined by CESTEH from studies involving populations non-exposed to pesticides, being 0.56 mmol/min/mg for AChE (for both genders) and 2.29 and 1.61 mmol/min/mg for BChE, for men and women, respectively [31]. Cholinesterase activity was considered normal when subjects presented values above the reference values.

### 2.3. Respiratory Health Assessment

Prevalence of respiratory symptoms was assessed by a questionnaire-guided interview, using the European Community Respiratory Health Survey (ECRHS), validated in Brazil by Ribeiro et al. [32]. This questionnaire evaluates respiratory symptoms in the previous 12 months. However, in the off-season period, it was adapted to identify the symptoms prevalence in the previous 4 months, in order to avoid overlapping with the crop season.

Spirometry was performed following the recommendations of the ATS/ERS—American Thoracic Society and the European Respiratory Society [33] with a Koko PFT spirometer (nSpire Health, Longmont, CO, EUA), calibrated daily. We used reference values proposed by Polgar and Promadhat [34] for males up to 24 years and females up to 20 years old and by Pereira et al. [35] for males aged over 25 years and females over 21 years old. Although only the latter set was derived from the Brazilian population, both are recommended by the Brazilian Thoracic Society [36]. In the crop season, the respiratory assessment was conducted one week after the first interview due to exam preparation.

This study focused on the forced vital capacity (FVC), the forced expiratory volume in the first second (FEV_1_), the FEV_1_/FVC ratio, and the forced expiratory flow between 25–75% (FEF_25–75%_). Individuals with the FEV_1_/FVC ratio below the predicted lower limit were classified as having an obstructive defect (OD). In these cases, severity classification was measured according to the FEV_1_ value in relation to the predicted: mild (FEV_1_ > 60%), moderate (FEV_1_ > 41 < 60%) and severe (FEV_1_ ≤ 40%). A restrictive pattern (RP) was defined for cases with simultaneous reduction of FVC and FEV_1_, but with FEV_1_/FVC ratio within the predicted range associated with at least one of the following (i) FVC reduction to levels below 50% of predicted value or (ii) presence of FEF_25–75%_/FVC ratio above 150% of predicted that may characterize increased intermediate expiratory flows, due to a rise in elastic recoil traction of lungs. Altered cases that did not meet the criteria for definition as an obstructive defect or restrictive pattern were classified as a nonspecific pattern (NSP) [33,37].

### 2.4. Statistical Analysis

Depending on their distribution, data were presented as mean and standard deviation (SD) or median and interquartile range (IQR). Comparison between groups was performed using T-test or Mann-Whitney test and comparison between categorical variables was performed using Chi-Square test. The associations of independent variables (including the IEB) and variables such as lung function results, AChE and BChE were evaluated in a regression analysis using Generalized Linear Models (GLM) [38]. Variables with *p* < 0.10 in the univariate analysis were considered for the multiple models. The GLM was fitted using the log-link function and Poisson scale response. Akaike’s Information Criterion (AIC) was applied to indicate the best fitting model. All models tested were controlled for smoking and age. Gender, weight and height were considered to establish the predicted values for spirometry. Socioeconomic status was similar among all participants and not included in the analysis. Each person was compared to himself for the presence of respiratory symptoms during the crop season and off-season, and the Odds Ratio was calculated through the McNemar test, as participants formed a matched control case. Statistical analysis was performed using IBM SPSS software (version 22 IBM Corp., Chicago, IL, USA). *p*-values < 0.05 were considered significant.

## 3. Results

### 3.1. Study Population

Some of the 82 participants in the exposure assessment refused to participate in the cholinesterase test, or their samples were insufficient, remaining 74 (90.2%) valid blood samples for analysis. Spirometry was performed in 70 (85.4%) individuals during the crop season and 62 (75.6%) in the off-season. Seventy-five (91.5%) individuals answered to the respiratory symptoms questionnaire in both periods.

Table 1 presents sociodemographic characteristics of the studied population classified by exposure group. Most rural workers were men (83.3%) with a mean age of 42.9 ± 12.4 (sd) and relatives were mostly women (85.3%) aging 45.7 ± 14.9 (sd) on average. They were predominantly married and, as many individuals in relatives’ group were married to participant rural workers, family income was similar among groups (up to two Brazilian minimum wages, approximately US $520). In general, 86.6% had primary or lower educational level, and only 13.4% had studied more than eight years. Most rural workers (60.4%) and relatives (76.5%) had never smoked, whereas 20.8% and 17.6% were ex-smokers, and only 18.8% and 5.9% were smokers at the time of data collection, respectively. Although the number of current smokers was higher among rural workers, the non-statistical significance may be due to the small size of the subgroups. Among rural workers and relatives, respectively, 50% and 38.2% had low or normal weight, 41.7% and 29.4% were overweight, and 8.3% and 32.4% were obese.

### 3.2. Exposure Assessment

Table 1 shows pesticide exposure characteristics of participants according to group of exposure. The length of pesticide exposure was long for both groups. Rural workers had a mean age of 42.9 years and a length of pesticide exposure of 30.2 ± 13.6 (sd) years, with an average of 10.7 ± 2.3 (sd) hours worked per day in the crop season. Among relatives, the mean age was 45.7 years, and the duration of exposure was 19.3 ± 15.1 (sd) years. Significantly fewer relatives stated to have direct contact with pesticide in the crop (*n* = 4; 11.8%) and to frequently handle pesticides (*n* = 3; 14.7%) at the time of data collection. Nevertheless, 29 (85.3%) relatives have claimed to assist in agricultural activities in the crop season as re-entry workers although only 10 (29.4%) declared to use any PPE. Thirty-eight rural workers (79.2%) reported handling and spraying pesticides by manual pumping or backpack tank more than once a week, and five of them (11.9%) used pesticides 4 to 7 times per week. About 75% of rural workers affirmed to wear respiratory protection, gloves and boots, but only 23% claimed to use eyes protection while applying pesticides.

Most individuals (53.7%) lived up to 500 m from a planting site. Forty rural workers (83.3%) and thirty-two relatives (94.1%) were domestically exposed to pesticides by using them at home or washing contaminated clothes and equipment (Table 1). Only 22.9% of rural workers and none relative were trained or received technical support to handle pesticides. Most of the rural workers (95.8%) and relatives (73.5%) consumed food and water on the crop site, including when pesticides were applied.

All participants presented values of AChE above the reference values, considered normal. Twelve out of 44 rural workers (27.3%) and 2 out of 30 relatives (6.7%) presented BChE levels below the reference values, considered abnormal. In the multiple regression models, AChE reduction pattern was significantly associated with the pesticide manipulation frequency (*p* = 0.04), whereas BChE presented an association with it near the significance level (*p* = 0.08). 

Subjects declared using regularly 49 pesticides from 31 chemical groups, including organophosphates, carbamates, pyrethroids, nitriles, diamides, neonicotinoids, avermectins and benzimidazole. Glyphosate, classified as highly toxic, was mentioned by 35.4% of rural workers, and paraquat, moderately toxic to humans, by 16.7%, while 6.3% reported using both. These products are banned in Brazil for tomato cultivation [1]. The use of other 15 extremely toxic pesticides and seven highly toxic ones were mentioned 91 and 36 times, respectively. Moreover, 21 moderately toxic pesticides were mentioned 81 times and five low toxicity pesticides were mentioned nine times. In addition, spraying Lorsban (chlorpyrifos), an extremely toxic organophosphate, was mentioned three times, and 2,4-D (aryloxyalkanoic acid), an extremely toxic herbicide, was mentioned once. Both are not permitted for tomato cultivation. Furthermore, the use of endosulfan, a highly toxic insecticide/acaricide prohibited in Brazil since 2013, was cited by one farmer [1].

### 3.3. Respiratory Health Assessment

Considering all participants, 33.3% reported none respiratory symptom in the crop season and 66% in the off-season. During the crop season, 32% of the interviewees had one and 22.7% two respiratory symptoms, whereas during the off-season, 18.7% had one symptom and 13.3% two symptoms. In both periods, the most prevalent symptoms were cough, nasal allergies and hay fever, chest tightness, and breathlessness. During the crop season and off-season, respectively, the prevalence was 40% and 30.7% for cough, 30.7% and 24% for nasal allergies and hay fever, 24% and 17.3% for chest tightness, and 17.3% and 10.7% for breathlessness. Among rural workers, 37% and 19.6% presented one and two symptoms during the crop season, and 17.8% and 11.1% in the off-season, respectively. Whereas among relatives, 24.1% and 27.6% showed one and two symptoms during the crop season, and 20% and 16.7% in the off-season, respectively. Although there were no statistically significant differences between the periods, the number and prevalence of respiratory symptoms were higher during the crop season.

The individual comparison of respiratory symptoms between crop season and off-season is presented in Table 2. The chance of having symptoms during the crop season was significantly higher than during the off-season for two symptoms. Six individuals woke with breathlessness during the crop season but not during the off-season, whilst the opposite did not happen. Eleven individuals woke up with cough during the crop season but not during the off-season, while only two individuals had the opposite (OR = 5.5).

Both during crop and off-season, most individuals (80%) presented normal spirometry. Table 3 shows the spirometry associated patterns among rural workers and relatives assessed in SJU in both periods. The most common pattern of pulmonary change found was obstructive, followed by non-specific. During the crop season, five rural workers presented mild OD, one moderate OD, and two presented NSP. Moreover, three relatives presented mild OD, one presented RP, and two had NSP. During the off-season, five rural workers presented mild OD, one presented RP and three presented reduced vital capacity and FEV_1_ close to inferior normal limit with normal FEV_1_/FVC ratio. Also, one relative presented mild OD and three presented NSP.

**Figure 1:**
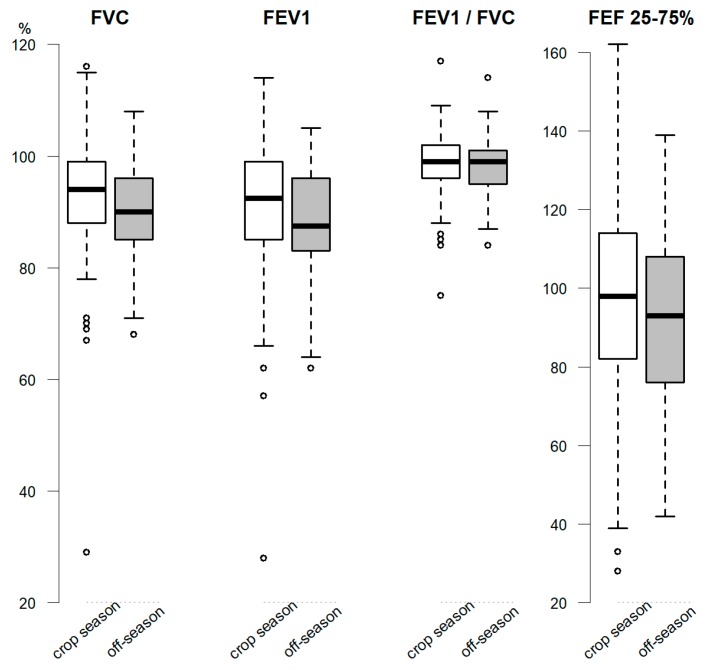
Boxplot of spirometry results comparison (in percentage of predicted) between crop season (2014) and off-season (2015) among family farmers in SJU. Notes: Light and dark boxes represent the crop season and off-season, respectively. The boxes show the interquartile range (IQR, 25th–75th percentile) and the horizontal line inside the box represents the median; the circles show the outlier values. FVC, FEV_1_ and FEV_1_/FVC presented non-normal distribution while FEF_25–75%_ presented normal distribution.

For each spirometry variable, the lower and upper limits, interquartile ranges, outliers, mean or median, were calculated and presented in boxplot in Figure 1. Non-statistical significant difference was seen in the comparison of evaluated periods. Nonetheless, values presented a slight reduction and less negative outliers during the off-season.

The multiple regression models show that spirometric variables are influenced by the proposed exposure indicators. Table 4 presents the association of the spirometry variables and cholinesterase enzymes with the exposure indicators during the crop season. FVC was associated with the years of working with pesticides as a rural worker or helper, and having two or more respiratory symptoms. FEV_1_ was related to the IEB, having two or more symptoms, and years of working with pesticide. FEV_1_/FVC was related to the frequency of handling pesticides, and to the IEB. FEF_25–75%_ was associated with the manipulation frequency, years of rural work, and having two or more respiratory symptoms.

Table 5 shows the association of the pesticide exposure indicators with the spirometry measures during the off-season in SJU. FVC was related to having two or more respiratory symptoms. FEV_1_ was associated with the manipulation frequency, and the years of working with pesticide as a rural worker or helper. FEV_1_/FVC ratio was related to the years of rural work, whilst presented an association near the significance level with the IEB. Moreover, FEF_25–75%_ was associated with the manipulation frequency, and years working with pesticide or helping as a rural worker.

## 4. Discussion

Only a few studies have been conducted in Brazil to access the health impacts among pesticide-exposed populations. An innovative approach of this study was to compare the respiratory effects of high and low-exposure periods. Our findings demonstrate an increased prevalence of self-reported cough, nasal allergies and hay fever, chest tightness, and breathlessness among workers and relatives, especially during the crop season. Furthermore, we found significant associations between the short and long-term exposure to pesticides and a decrease in lung function parameters in both crop season and off-season. These findings provide complementary evidence of the acute and chronic effects of pesticide exposure on respiratory health and possibly the development of chronic lung diseases.

We found a significant association between some exposure indicators used and a decrease of FVC, FEV_1_, FEV_1_/FVC ratio, and FEF_25–75%_ both during the crop season and off-season, even after adjusting for sex, age and smoking. In general, more exposure indicators were significantly related with the lung measures. Moreover, some associations were stronger during the crop season, suggesting that short-term exposure to pesticides had an additional effect on spirometry parameters. Changes in FEV_1_ and FEV_1_/FVC ratio are predominantly related to large airways, and FEF_25–75%_ alterations to small airways [39]. Further studies should be done in SJU to investigate the specific segment affected. Previous studies investigated the pulmonary function of pesticide-exposed workers and found a significant decrease in the FVC [8,9], in the FEV_1_ [8,9,17,18,19,20,40,41], in the FEV_1_/FVC ratio [9,11,17,20], in the FEF_25–75%_ [8,9,18,40], and in the peak expiratory flow [9,17]. In addition, the only study that evaluated the influence of pesticide exposure on the lung function of Brazilian rural workers found a prevalence of obstructive diseases higher than our findings [23]. Taken together, these studies reinforce the association between respiratory impairments and occupational exposure to pesticides, independent of smoking.

Not many studies have discussed the respiratory effects of pesticide exposure considering seasonal variations. In the present study, we did not find statistically significant differences on lung function when comparing the crop season and the off-season. Nevertheless, the spirometry variables presented a slight reduction during the off-season, in accordance with another study [19]. This minor reduction could be explained by the worsening of the individual condition, by less effort of the participants at the reevaluation tests or by loss of follow-up subjects at this stage, especially those in better health condition. Previous studies found a significant reduction in the FEV_1_ measurement in the post-exposure when compared to the pre-exposure level [42], and lower post-shift values of FVC and FEV_1_ in both crop- and off-season [19]. It suggests that acute obstructive diseases can arise from high exposure in crop activities. Even when all standards and recommendations are followed, a precise estimation of the individual spirometric changes requires a relatively prolonged follow-up due to seasonal, technical and biological variability [19]. Unfortunately in our study this was not possible.

In this study, the higher prevalence of respiratory symptoms during the crop season can be attributable to the short-term effects of pesticide exposure. These findings are supported by several epidemiological studies that associated respiratory symptoms to occupational pesticide exposure in Brazil [21,22,23] and elsewhere [8,9,13,17,29].

In Brazil, family farmers frequently handle multiple pesticides and apply them by manual pumping or backpack tanks. Nonetheless, pesticide exposure is not restricted to direct contact during the preparation and spraying. Commonly, rural workers are involved in all stages of the cultivation process and very often are helped by their relatives in different agricultural tasks during the crop season [1,3,21]. Although many relatives do not often participate directly in spraying activities, they are occupationally exposed to pesticides when helping in other tasks, such as taking the sprout out, tying the stems or harvesting the tomatoes. Some of these activities are carried out at the same day or day after the pesticide spraying and are often done without personal protection. This situation was observed in SJU. Moreover, our findings corroborate previous studies conducted in Brazil which have shown that, in general, family farmers present low educational level and family income, and lack of orientation or technical support for using chemical products. This scenario points to social vulnerability, leading to a low risk-awareness and a misuse of protective equipment and, consequently, to careless pesticide use and higher human exposure [3,21,22,23,43]. Indeed, during the field work in SJU it was observed that no one used complete PPE even during spraying activities. In the interviews they identified this equipment as expensive, hot and uncomfortable. Although most rural workers affirmed that they shower and wash their hands after handling pesticides, this only occurs at the end of the work day. Most also declared consuming food and water in the field during work. These habits may increase the exposure and contamination risk [3,44]. Furthermore, some highly toxic pesticides such as glyphosate-based herbicides, paraquat, lorsban, and 2,4-D, that are banned for tomato cultivation in Brazil, were used in SJU.

Family farmers in SJU, besides occupational exposure, are often environmentally exposed to pesticides from an early age, either by living near planting sites, by using or storing pesticides at home, or by having contact with contaminated clothes and work tools. This residential contact can represent an extra pesticide exposure to rural families and increment the risk and effects on human health [1,44].

The Brazilian law states that all agricultural workers must be submitted to periodic medical examinations with cholinesterase measurements, however, these are not provided by public health services. The exposure assessment and health care of family farmers in Brazil are limited by the informal organization of these workers and their distribution in approximately 4.3 million small properties [45], the constant and prolonged exposure to low doses of multiple pesticides, the distance to health services, the shortage of laboratories with available analytical capacity, and the absence of an integrated intoxication reporting system [1,4]. The AChE and BChE activities vary widely among population groups but their reduction may indicate chronic and acute exposures, respectively [3]. Although the participants were exposed to multiple pesticides in SJU, a significant relation was observed only between AChE inhibition and manipulation frequency, and few individuals presented BChE below the proposed reference values. This can be partially explained by the chosen reference values, by the sample size, or because these biomarkers reflect only the exposure to a small portion of the pesticides used in SJU. AChE has been pointed to be inadequate for monitoring low-dose chronic exposure [46,47], and the BChE reboot effect and fast recovery can hide or underestimate unsafe pesticides exposure [44], making their use as a biomarker for pesticide exposure controversial. Nevertheless, the relation between pesticide exposure and the cholinesterase depletion has been reported in longitudinal studies [42,48,49] and in cross-sectional ones comparing to non-exposed controls [3,9,44]. Agricultural workers in India presented an AChE inhibition of 34.2% and positive associations with respiratory symptoms, lung function decrement and COPD, compared to controls [9]. A study conducted in Brazil showed that, compared to unexposed controls, rural workers and rural area residents presented BChE depletion during the exposure period and AChE depression during both the exposure and non-exposure periods. On the other hand, 31.7% had AChE over 30% higher than baseline levels, indicating a reboot effect [44]. As limitations of our study we can point out the fact that a few individuals did not participate at all stages, the lack of urine biomarkers and the absence of an unexposed control group. The years of rural work were not considered in the IEB because the frequency of manipulation was more explanatory in the analysis. However, the years of working or helping as a rural worker presented significant relations to the pulmonary function impairments in the multiple regression models.

The strong points of this research are: (a) it included approximately 11% of tomato growers in the municipality; (b) it adopted an important methodological approach based on multiple sources of evidences collected by a large multidisciplinary team in two seasons of the year; (c) the assessment of seasonal variations; (d) it presented an exposure burden measurement which, even in a small sample size, was associated with respiratory impairments and could be replicated, or even improved, in other studies; (e) the focus on family farmers, which are responsible for most of the food produced in Brazil; (f) the consideration of rural workers and their relatives as exposed groups; and (g) the broader view of the pesticide exposure, considering the residential distance to agricultural areas.

## 5. Conclusions

This study reinforces previous evidence that short or long-term exposures to pesticides are associated with a clinically relevant prevalence of respiratory symptoms and pulmonary function impairment among family farmers often exposed occupationally and environmentally. This situation deserves special attention and urgent preventive measures as poor respiratory condition at productive age may decrease the quality of life of adults and elderly and increase the risk of chronic disease. A higher morbidity and mortality burden associated with these diseases impacts the health system and increases costs. Understanding the family farmers’ health situation is essential to establish early diagnosis, and offer appropriate treatments and preventive measures.

Brazil is the world largest consumer of pesticides but local evidences of their impacts are very scarce and further research is much needed. This study helps to show that occupational exposure to pesticides can culminate in adverse respiratory health outcomes in family farmers and reinforces the need for adoption of more personal protection measures and sustainable agricultural practices.

Despite this research being conducted in a small rural community in Brazil, similar situations are very common in family farming and widespread in most of the low- and middle-income countries. Moreover, data produced reinforces causal relationships and can help the design of effective intervention measures and public policies to reduce exposure, risks and the consequences for human health and the environment.

## Figures and Tables

**Table 1:** Sociodemographic and pesticide exposure characteristics of a rural population in SJU, according to groups of exposure.

Sociodemographic Variables	Total *n =* 82 (%)	Rural Workers *n =* 48 (%)	Relatives *n =* 34 (%)	*p*-Value
Age (mean in years ± sd)	44.0 ± 13.5	42.9 ± 12.4	45.7 ± 14.9	0.35 ^b^
Gender				
Male	45 (54.9%)	40 (83.3%)	5 (14.7%)	<0.001 ^b^
Female	37 (45.1%)	8 (16.7%)	29 (85.3%)	
Marital status				
Married/cohabiting partner	71 (86.6%)	40 (83.3%)	31 (91.2%)	0.31 ^b^
Single or divorced	11 (13.4%)	8 (16.7%)	3 (8.8%)	
Monthly family income ^$^				
Up to 2 salaries	58 (70.7%)	37 (77.1%)	21 (61.8%)	0.133 ^b^
More than 2 salaries	24 (29.3%)	11 (22.9%)	13 (38.2%)	
Years of education (median; IQR *)	4 (3.3–8)	4 (3–6.5)	5 (4–8)	0.30 ^c^
Body Mass Index (mean ± sd)	26.4 ± 5.6	25 ± 4	28.5 ± 6.9	0.48 ^c^
Low or normal weight	37 (45.1%)	24 (50%)	13 (38.2%)	0.021 ^b^
Overweight	30 (36.6%)	20 (41.7%)	10 (29.4%)	
Obese	15 (18.3%)	4 (8.3%)	11 (32.4%)	
Smoking status				
Never	55 (67.1%)	29 (60.4%)	26 (76.5%)	0.16 ^a^
Past (ex)	16 (19.5%)	10 (20.8%)	6 (17.6%)	0.78 ^a^
Current	11 (13.4%)	9 (18.8%)	2 (5.9%)	0.11 ^a^
Mean/Median (pack-years)	2.8/0	6.4/0	1.0/0	0.09 ^c^
Alcohol consumption (if yes)	24 (29.3%)	14 (29.2%)	10 (29.4%)	1.00 ^a^
Exposure variables				
Duration of pesticide exposure (mean in years ± sd)	25.7 ± 15.1	30.2 ± 13.6	19.3 ± 15.1	0.001 ^b^
Current direct contact in the crop (if yes)	42 (51.2%)	38 (79.2%)	4 (11.8%)	<0.001 ^a^
Frequent handling in the crop season **	41 (50%)	38 (79.2%)	3 (8.8%)	<0.001 ^a^
Pesticide use in the off-season	6 (7.3%)	6 (12.5%)	0	0.03 ^a^
Use of any PPE ***	53 (64.6%)	43 (89.6%)	10 (29.4%)	<0.001 ^a^
Use of respiratory PPE (mask or respirator)	39 (47.6%)	37 (77.1%)	2 (5.9%)	<0.001 ^b^
Use of eyes PPE (visor)	11 (13.4%)	11 (22.9%)	0	0.003 ^b^
Use of hand PPE (gloves)	41 (50%)	35 (72.9%)	6 (17.6%)	<0.001 ^b^
Use of shoes PPE (boots)	42 (51.2%)	36 (75%)	6 (17.6%)	<0.001 ^b^
Domestic exposure (if yes)	72 (87.8%)	40 (83.3%)	32 (94.1%)	0.141 ^a^
Residential distance from plantation site				
Up to 500 m	44 (53.7%)	23 (47.9%)	21 (61.8%)	0.215 ^b^
More than 500 m	38 (46.3%)	25 (52.1%)	13 (38.2%)	
Previous intoxication ever	14 (17.1%)	11 (22.9%)	3 (8.8%)	0.095 ^a^
Received training or technical support	11 (13.4%)	11 (22.9%)	0 (0%)	0.003 ^a^
Washes hands after handling pesticides	63 (76.8%)	42 (87.5%)	21 (61.8%)	0.007 ^a^
Takes shower after handling pesticides	47 (57.3%)	33 (68.8%)	14 (41.2%)	0.013 ^a^
Consumes food and water in the field	71 (86.6%)	46 (95.8%)	25 (73.5%)	0.004 ^a^

^$^ Based on Brazilian minimum salary (±293 U.S. $); * IQR—Interquartil range (P25–P75); ** Frequent pesticide handling = more than 1 to 3 times per week; *** PPE: Personal protective equipment; ^a^ Chi-Square- Fisher exact test; ^b^ One-way ANOVA; ^c^ Kruskal-Wallis one-way ANOVA.

**Table 2:** Comparison of respiratory symptoms prevalence between crop season (2014) and off-season (2015), using the ECRHS questionnaire in SJU.

Symptoms	Crop Season/Off-Season Periods ^a^				Odds Ratio (95% CI) ^b^	*p*-Value
	Yes/No	No/Yes	Yes/Yes	No/No		
Wheeze or chest tightness	9	4	6	51	2.25 (0.63, 10)	0.27
Wheeze with breathlessness	2	3	3	62	0.67 (0.06, 5.82)	1
Wheeze without cold	3	2	3	62	1.5 (0.17, 17.96)	1
Waking with chest tightness	6	1	7	56	6 (0.73, 275.99)	0.13
Waking with breathlessness	6	0	5	59	Not calculable	0.04 *
Waking with cough	11	2	19	38	5.5 (1.20, 51.07)	0.03 *
Asthma crisis	2	1	0	67	2 (0.1, 118.10)	1
Nasal allergies and hay fever	12	5	10	43	2.4 (0.79, 8.70)	0.15
Treatment for asthma	0	2	1	67	0 (0, 5.32)	0.48
Asthma diagnosis ^c^	1	3	1	65	0.33 (0.01, 4.15)	0.62

^a^ Comparison between crop season and off-season periods, being ‘*Yes*’ for ‘*with symptoms*’ and ‘*No*’ for ‘*without symptoms*’; ^b^ Odds Ratio calculated through McNemar test and Confidence Interval (CI) = 95%; ^c^ Asthma diagnosis = at least one asthma attack in the past 12 months and/or confirmation of medication use. * Values with statistical significance.

**Table 3:** Spirometry patterns among individuals assessed in SJU during crop season and off-season.

Spirometry Patterns	Crop Season			Off-Season		
	Rural Workers (*n* = 43)	Relatives (*n* = 27)	Total (*n* = 70)	Rural Workers (*n* = 38)	Relatives (*n* = 24)	Total (*n* = 62)
Normal	35 (81.3%)	21 (77.8%)	56 (80%)	29 (76.3%) *	20 (83.3%)	49 (79%)
OD ^1^	6 (14%)	3 (11.1%)	9 (12.9%)	5 (13.2%)	1 (4.2%)	6 (9.7%)
RP ^2^	0	1 (3.7%)	1 (1.4%)	1 (2.6%)	0	1 (1.6%)
NSP ^3^	2 (4.7%)	2 (7.4%)	4 (5.7%)	0	3 (12.5%)	3 (4.8%)

^1^ OD: obstructive disease; ^2^ RP: restrictive pattern; ^3^ NSP: non-specific pattern; * 3 rural workers presented vital capacity and FEV_1_ close to inferior normal limit with normal FEV_1_/FVC ratio.

**Table 4:** Multiple regression models ^a^ of spirometry variables (in percentages of predict) and cholinesterase levels on exposure indicators during the crop season in SJU. 2014.

Variables	Exposure Indicators	β-Coefficient (CI) ^b^	*p*-Value
FVC	Years of rural work ^c^	−0.01 (−0.28; −0.14)	<0.001
	Symptoms ^d^	−0.79 (−1.21; −0.04)	0.005
FEV_1_	IEB ^e^	−0.06 (−0.09; −0.023)	0.001
	Symptoms ^d^	−0.11 (−0.17; −0.05)	<0.001
	Years of rural work ^c^	−0.003 (−0.005; −0.002)	0.01
FEV_1_/FVC	Manipulation frequency	−0.85 (−1.74; −0.89)	<0.001
	IEB ^e^	−0.11 (−1.05; 0.13)	0.05
FEF_25–75%_	Manipulation frequency	−0.62 (−0.77; −0.48)	<0.001
	Years of rural work ^c^	−0.05 (−0.07; −0.03)	<0.001
	Symptoms ^d^	−0.89 (−1.14; −0.36)	0.002
AChE	Manipulation frequency	−14.27 (−27.11; −1.44)	0.039
BChE	Manipulation frequency	−11.80 (−25.24; −1.64)	0.08

^a^ multiple analysis adjusted for age and smoking; ^b^ confidence interval = 95%; ^c^ years of working or helping as rural worker; ^d^ two or more declared respiratory symptoms; ^e^ IEB: individual exposure burden.

**Table 5:** Multiple regression models ^a^ of spirometry variables (in percentages of predict) on exposure indicators during the off-season in SJU. 2015.

Spirometry Variables	Exposure Indicators	β-Coefficient (CI) ^b^	*p*-Value
FVC	Symptoms ^d^	−0.79 (−1.21; −0.04)	0.005
FEV_1_	Manipulation frequency	−0.29 (−0.37; −0.28)	<0.001
	Years of rural work ^c^	−0.02 (−0.03; −0.009)	<0.001
FEV_1_/FVC	Years of rural work ^c^	−0.001 (−0.001; −0.001)	<0.001
	IEB ^e^	−0.001 (−0.002; 0.000)	0.07
FEF_25–75%_	Manipulation frequency	−0.34 (−0.42; −0.26)	<0.001
	Years of rural work ^c^	−0.03 (−0.04; −0.02)	<0.001

^a^ multiple analysis adjusted for age and smoking; ^b^ confidence interval = 95%; ^c^ years of working or helping as rural worker; ^d^ two or more declared respiratory symptoms; ^e^ IEB: individual exposure burden.
